# Inflammation and Neutrophil Oxidative Burst in a Family with *NFKB1* p.R157X LOF and Sterile Necrotizing Fasciitis

**DOI:** 10.1007/s10875-023-01461-3

**Published:** 2023-03-09

**Authors:** Wenny Santaniemi, Pirjo Åström, Virpi Glumoff, Nora Pernaa, Ella-Noora Tallgren, Sanna Palosaari, Antti Nissinen, Meri Kaustio, Outi Kuismin, Janna Saarela, Katariina Nurmi, Kari K. Eklund, Mikko R. J. Seppänen, Timo Hautala

**Affiliations:** 1 Research Unit of Biomedicine, University of Oulu, Oulu, Finland; 2 Biocenter Oulu, University of Oulu, Oulu, Finland; 3 Cancer and Translational Medicine Research Unit, University of Oulu, Oulu, Finland; 4 Institute for Molecular Medicine Finland, HiLIFE, University of Helsinki, Helsinki, Finland; 5 Department of Clinical Genetics, Oulu University Hospital, Oulu, Finland; 6 Centre for Molecular Medicine Norway, University of Oslo, Oslo, Norway; 7 Faculty of Medicine, Clinicum, Translational Immunology Program, University of Helsinki, Helsinki, Finland; 8 Department of Rheumatology, Inflammation Center, University of Helsinki and Helsinki University Hospital and Orton Orthopedic Hospital, Helsinki, Finland; 9 Adult Immunodeficiency Unit, Infectious Diseases, Inflammation Center, University of Helsinki and HUS Helsinki University Hospital, Helsinki, Finland; 10 Rare Disease Center and Pediatric Research Center, Children and Adolescents, University of Helsinki and HUS Helsinki University Hospital, Helsinki, Finland; 11 Infectious Diseases, Oulu University Hospital, Oulu, Finland

**Keywords:** Inflammation, Innate immunity, Neutrophils

## Abstract

Loss-of-function (LOF) mutations in *NFKB1*, coding for p105, may cause common variable immunodeficiency due to dysregulation of nuclear factor kappa-light-chain-enhancer of activated B cells (NF-κΒ) pathway. Monoallelic LOF variants of *NFKB1* can predispose to uncontrolled inflammation including sterile necrotizing fasciitis or pyoderma gangrenosum. In this study, we explored the impact of a heterozygous *NFKB1* c.C936T/p.R157X LOF variant on immunity in sterile fasciitis patients and their family members. The p50 or p105 protein levels were reduced in all variant carriers. Interleukin-1β (IL-1β) and interleukin-8 (IL-8) levels were elevated in vitro, potentially contributing to the very high neutrophil counts observed during fasciitis episodes. Phosphorylation of p65/RelA was reduced in p.R157X neutrophils suggesting defective activation of canonical NF-κB. Oxidative burst after NF-κB-independent phorbol 12-myristate 13-acetate (PMA) stimulation was similar in both p.R157X and control neutrophils. Comparable amounts of nicotinamide adenine dinucleotide phosphate (NADPH) oxidase complex subunits were found in p.R157X and control neutrophils. However, a compromised oxidative burst was observed in p.R157X neutrophils following activation of NF-κB-dependent mechanisms following stimulation of toll-like receptor 2 (TLR2) and Dectin-1. Neutrophil extracellular trap formation was not affected by p.R157X. In summary, the *NFKB1* c.C936T/p.R157X LOF variant has an impact on inflammation and neutrophil function and may play a role in the pathogenesis of sterile necrotizing fasciitis.

**Supplementary Information:**

The online version contains supplementary material available at 10.1007/s10875-023-01461-3.

## Introduction

Nuclear factor kappa-light-chain-enhancer of activated B cells (NF-κΒ) protein complex regulates the transcriptional activity in inflammation, with an impact on cytokine production, cell differentiation, and survival [1]. In the adaptive immune system, NF-κΒ pathway is activated in lymphocytes in response to various stimuli including microbial antigens, activation of pattern recognition receptors (PRR), and ligands of numerous cytokine receptors [2]. Innate immune cells including macrophages, dendritic cells, and neutrophils detect microbial components via PRRs, which then activate the canonical NF-κΒ pathway [2] responsible for the control of inflammasome activity [3, 4]. In neutrophils, NF-κΒ activity can be needed for the activation of oxidative burst [5, 6]. In experimental models, NF-κΒ deficient mice display spontaneous skin and soft tissue inflammation in association with high blood neutrophil counts [7]. In serious neutrophilic dermatoses, altered neutrophil recruitment and activation is observed [8, 9]. Neutrophil functions are essential for innate immunity, while their dysregulation may contribute to inflammatory disease processes [10]. However, the role of NF-κΒ signaling in the regulation of neutrophil function in necrotizing fasciitis is not well understood.

Mutations in NF-κΒ pathways may cause infection susceptibility, autoimmunity, and autoinflammation [1]. Most commonly, *NFKB1* loss-of-function (LOF) causes B cell deficiency in individuals suffering from common variable immunodeficiency (CVID) [11, 12]. *NFKB1* LOF may also lead to extreme pro-inflammatory cytokine production in association with necrotizing fasciitis or pyoderma gangrenosum [13–16]. We have previously described two family members with necrotizing fasciitis in association with a heterozygous *NFKB1* NM003998:c.469C > T p.R157X LOF mutation [13]. The patients suffered from recurrent non-bacterial necrotizing fasciitis, during which they presented with very high neutrophil counts [13]. In this study, we further characterize the adaptive and innate immunity in these two patients, as well as in the carriers of *NFKB1* p.R157X in their family in three generations. First, we studied inflammasome activation and cytokine production in these family members. We also examined the impact of p.R157X on Dectin-1 or Toll-like receptor 2 (TLR2) mediated NF-κΒ activation of neutrophils. We further examined if oxidative burst and neutrophil extracellular trap (NET) formation could be affected by p.R157X [2] [17] [4].

## Patients and Methods

### The NFKB1 p.R157X Family

**Fig. 1:**
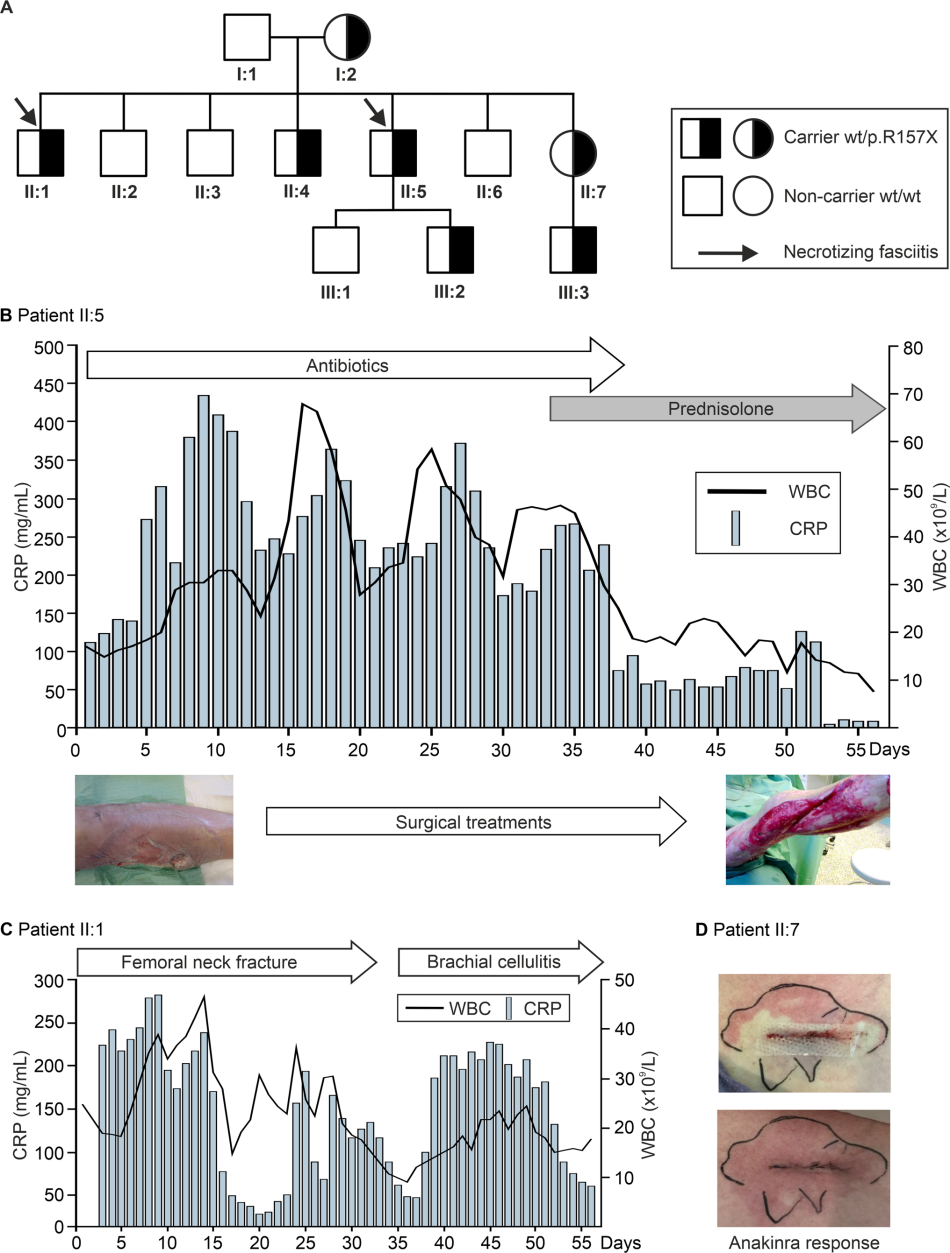
Family tree and clinical summary of inflammatory reactions in patients. Pedigree chart **A** shows symptomatic cases suffering from recurrent necrotizing fasciitis (arrows) and asymptomatic carriers (shaded) of *NFKB1* p.R157X variant. **B** Patient II:5 developed an episode of severe sterile necrotizing fasciitis with high C-reactive protein (CRP) levels and robust increases in white blood cell (WBC) counts. Administration of broad-spectrum antibiotics or prednisolone is shown. Clinical photographs before the first operative intervention and before skin grafting are shown in connection with the timeline. **C** Patient II:1 suffered from an episode of sterile necrotizing fasciitis during which 14 surgical revisions were completed. The patient displayed high CRP concentrations and a strongly elevated WBC count. **D** Wound photographs (patient II:7) before and after anakinra (Kineret**®**) administration on three consecutive days

We identified this family with *NFKB1* (NM_003998) c.C936T/p.R157X LOF mutation (Fig. 1A) based on highly unusual recurrent non-bacterial hyperinflammatory necrotizing fasciitis episodes [13]. These episodes were triggered by surgical operations and presented with extremely high blood neutrophil counts (Fig. 1B and C, patients II:5 and II:1). Patient II:7 developed a severe postoperative inflammatory skin reaction treated with anakinra (Fig. 1D). We extended the evaluation to their asymptomatic first-degree relatives (Fig. 1A). All family members gave informed consent. The study has been approved by Oulu University Hospital Ethical Committee.

### Patient I:2 (NFKB1 wt/p.R157X)

A healthy female with no history of infection susceptibility, autoinflammation or autoimmunity, who had received surgical treatment for rotator cuff rupture at the age of 70, cataract extraction at the age of 79 and urothelial carcinoma at the age of 84. At the age of 89, she had her first pneumonia episode, from which she recovered well. At the age of 90, she remained asymptomatic and had not suffered from any unusual infections.

### Patient II:1 (NFKB1 wt/p.R157X)

Childhood and young adulthood of this male patient were unremarkable. There was no previous clinical evidence of susceptibility to respiratory infections, autoinflammatory or autoimmunity features. His wound healing had been normal.

At the age of 48, the patient experienced a traumatic femoral neck fracture followed by prompt surgical treatment using mounting by cannulated screw (Asnis®, Stryker, Switzerland) (Fig. 1C). Two days later, edema and discharge from the wound was noted. A total of 14 surgical operations were required because of active inflammation and necrosis. All blood and surgical site bacterial and fungal cultures remained negative. C-reactive protein (CRP) levels and white blood cell (WBC) counts were elevated (Fig. 1C). He received broad-spectrum antibiotics (cefuroxime, piperacillin/tazobactam, ciprofloxacin, clindamycin, rifampin, vancomycin, and fluconazole in various combinations) without obvious benefits.

While recovering from this postoperative inflammatory episode, he developed another episode of skin and soft tissue inflammation. Brachial cellulitis and necrotizing fasciitis were diagnosed. After repeated surgical revisions, all microbiological samples again remained negative for bacteria or fungi. Inflammation accompanied by repeated corrective surgical procedures continued for the following year after these two acute episodes.

At the age of 56, the patient experienced a clavicular fracture with significant dislocation. Postoperative soft tissue necrosis with negative microbiology again led to repeated surgical revisions. He developed high postoperative fever with elevated CRP concentrations and WBC counts. Combinations of broad-spectrum antibiotics (piperacillin/tazobactam, vancomycin, linezolid, clindamycin) appeared ineffective.

### Patient II:5 (NFKB1 wt/p.R157X)

Childhood of this male patient was unremarkable without evidence of infection susceptibility, autoinflammation or autoimmunity. His wound healing appeared normal in childhood. Examples of clinical events are summarized in Fig. 1B.

At the age of 28, he experienced recurrent dislocation of his right patella leading to surgical treatment. Four days after the operation, he developed postoperative necrosis. At admission, a large subcutaneous abscess was evacuated. He then required repeated operations and intensive care. Microbiological analyses of all blood and surgical site samples remained negative. Rapidly, the patient developed bilateral gluteal abscesses followed by severe inflammation involving his entire right lower limb. Repeated surgical procedures and broad-spectrum antibacterial and antifungal treatments were attempted to control the tissue necrosis, after which a series of reconstructive operations and intense rehabilitation were required.

At the age of 36, he experienced minor skin injury to his ankle, followed by complicated tissue inflammation and culture-negative subcutaneous abscesses. Again, hospitalization, intravenous antibiotics, and repeated revisions were required. At the age of 39, after surgical removal of lipoma on the left scapular region was followed by local, uncomplicated inflammation of the surgical site. At the age of 41, a ruptured left rotator cuff tendon was operated. Soon thereafter, he developed local abscesses evacuated surgically. Again, all microbiological analyses remained negative.

At the age of 46, he developed an abscess and cellulitis of his left knee following a minor injury. After repeated surgical drainage and reconstructive surgery, he developed chest cellulitis necessitating the partial removal of skin and subcutaneous tissues. All tissue and blood microbiological samples remained negative. Blood concentrations of CRP and WBC counts were abnormally high (Fig. 1B). Oral prednisolone was effective to control inflammation.

### Patient II:7 (NFKB1 wt/p.R157X)

This female patient experienced several uncomplicated respiratory infections at preschool age. Since then, she has not suffered from infection susceptibility, autoimmunity or autoinflammation. She had been operated for appendicitis at the age of 19, with uneventful recovery. At the age of 35, an umbilical hernia was successfully operated. At the age of 53, she underwent a successful extraction of intervertebral disc protrusion. At the age of 63, breast cancer operation was uneventful. However, the removal of a benign skin lesion led to significant postoperative wound inflammation at the age of 64. By then, a known carrier of p.R157X, she received anakinra (Kineret®) 100 mg injections on three consecutive days (Fig. 1D). Thereafter, her recovery was prompt and uneventful.

### Patient III:2 (NFKB1 wt/p.R157X)

Patient III:2 suffered from episodes of tonsillitis and purulent otitis media in his childhood. Tonsillectomy was performed at the age of 18. He experienced an episode of viral pericarditis at the age of 31. At the age of 34, he accidentally developed a knee injury. However, surgical treatment was not needed, and he recovered well.

### Patient III:3 (NFKB1 wt/p.R157X)

This male patient was healthy until the age of 8, when he developed juvenile insulin-dependent diabetes. By the age of 24, he had not developed any complications or infection susceptibility.

### Other Family Members

Other family members (*NFKB1* wt/wt I:1, II:2, II:3, II:6, III:1) and (*NFKB1* wt/p.R157X II:4) had no clinical or laboratory evidence of infection susceptibility, autoinflammation or autoimmunity.

### Genetic Analysis

Exome and targeted Sanger sequencing were completed to index brothers with sterile necrotizing fasciitis and detected the NM003998:c.469C > T p.R157X stop-gain mutation. Parents, siblings, and their children were screened for p.R157X as described earlier [13].

### Isolation of Peripheral Blood Mononuclear Cells (PBMC) and Neutrophils

PBMCs were isolated by Ficoll-Paque gradient centrifugation from lithium heparin tubes or BD vacutainer cell preparation tubes (CPT, Becton–Dickinson) and stored at − 140 °C. Before any experiment, the cells were cultured overnight at 37 °C with 5% CO_2_, and 95% humidity.

Neutrophils were isolated from venous blood collected to lithium heparin tubes. The blood was mixed 1:1 to 3% Dextran solution with 154 mM NaCl and allowed to stand for 20 min at room temperature (RT). The top layer with neutrophils was transferred into a new tube and centrifuged for 10 min, 500 g at RT. The cell pellet was resuspended into 10 mL of PBS, then pipetted on top of Ficoll and separated at 400 g for 40 min at RT. The granulocyte layer was transferred into a new tube. Red blood cells were lysed with 5 mL of sterile water for 28 s. The lysis was stopped with an equal volume of 2 × PBS. Finally, the neutrophils were collected by centrifuging 500 g for 5 min RT and resuspended into the RPMI medium containing 2 mM L-glutamine.

### Western Blotting

The proteins were extracted to cell lysis buffer (9803S; Cell Signaling Technologies) according to the manufacturer’s protocol in the presence of Halt™ Protease Inhibitor cocktail and phosphatase inhibitors (Thermo Fisher Scientific). Protein concentrations were measured with DC protein assay (Bio-Rad Laboratories). Ten micrograms of proteins in the Laemmli sample buffer (BioRad) were separated in reducing conditions on a 10% sodium dodecyl-sulfate polyacrylamide gel and transferred onto Immobilon-FL PVDF membrane (Millipore). After blocking with Odyssey® blocking buffer (LI-COR), 0.17 µg/mL rabbit anti-NFKB1 (#3035; Cell Signaling Technologies) or 1 µg/mL mouse anti-GAPDH (Clone 258; Thermo Fisher Scientific) antibodies were allowed to bind overnight at 4 °C. After 3 × 5 min washes with Tris-buffered saline/0.1% Tween-20 (TBS-T) 1:10 000 anti-mouse or anti-rabbit IRDye 680RD or 800CW secondary antibodies (LI-COR) were added for 1 h at RT. Finally, after washing as above, the immune complexes were detected by Odyssey infrared scanner (LI-COR) and the intensities were analyzed by Image Studio Lite Ver 5.2 (LI-COR).

### NADPH Oxidase Complex Subunit Analysis

Protein extraction, Western blotting, RNA isolation, and quantitative PCR methods to examine the NADPH oxidase complex subunits (gp91^phox^, p22^phox^, p40^phox^, p47^phox^, p67^phox^, RAC2) are described in detail in Supplement Methods.

### RelA/p65 Phosphorylation

Venous blood was collected to lithium heparin tubes and 10 zymosan particles per cell (138 µg/ml) were added at RT for 1, 20, and 40 min in duplicates. For a non-stimulated sample, only a buffer was added. The samples were placed on ice and 1 ml of preheated BD Phosflow™ Lyse/Fix Buffer was added. After vortexing, the samples were incubated for 10 min at 37 °C, vortexed again and placed on ice. After centrifugation at 400 g for 8 min at 4 °C, the samples were washed twice with PBS and fixed with methanol for 10 min on ice. 1.5 mL of FACS buffer (0.5% bovine serum albumin in PBS) was added to each sample. After washing twice, the samples were resuspended into the FACS buffer. The cells were then stained with NF-κB-p65 (pSer529) and CD66b antibodies for 30 min in dark at RT (BD Biosciences). After two washes, the samples were measured with BD LSRFortessa™ using BD FACSDiva software and analyzed with FlowJo™ 10 software (BD Biosciences).

### Neutrophil Oxidative Burst

First, nitro blue tetrazolium test (NBT) was used to screen for a possibility of chronic granulomatous disease as described [18, 19]. Second, the granulocyte oxidative burst was analyzed using dihydrorhodamine (DHR) assay [20]. To stimulate the NF-κB dependent Dectin-1 and TLR-2 receptor signaling, zymosan (+ / − opsonization according to the manufacturer’s protocol; BioParticles®, Molecular probes) was added to 400 µL of fresh whole blood to the final concentration of 50 × 10^3^ particles/µl (138 µg/ml) for 1 h at RT. A 10 × volume of warm DHR-hemolysis solution containing 1.55 M NH_4_Cl, 0.1 M NaHCO_3_, and 10 mM of EDTA; pH 8 was added and the samples were incubated for 5 min at 37 °C. The cells were collected by centrifuging 200 g for 5 min and washed with Hank’s Balanced Salt Solution (HBSS), without Ca^2+^, Mg^2+^, or phenol red and with additional 0.1% (m/V) human albumin fraction V and 1 mM of EDTA, pH 8. After washing, the cells were incubated in HBSS containing DHR 123 (Sigma-Aldrich) and catalase in final concentration of 1.026 mM and 1.79 nM respectively for 5 min at 37 °C. As a positive control for reactive oxygen species (ROS) production, zymosan pre-incubation was replaced by adding PMA into final concentration of 1.6 µM. For negative control, HBSS-buffer was added. The samples were incubated for 14 min at 37 °C, vortexed, filtered, and analyzed immediately by flow cytometry (BD LSRFortessa, BD Biosciences, San Jose, CA). 4′,6-diamidino-2-phenylindole (DAPI; Invitrogen) was used to exclude dead cells.

### Neutrophil Extracellular Trap Formation (NETosis)

Cytotox Green dye (Essen BioScience) was added to freshly isolated neutrophils (400,000 cells/mL) in final concentration of 250 nM. In 50 µl, 2 × 10^4^ cells were added into wells of 96-well image lock plate and equal volume of 100 µg/mL zymosan or medium as negative control was added (eight wells per condition). PMA (100 nM final concentration) effect on NETOsis was first used as a positive control (Supplemental Fig. 4 and 5). The cells were analyzed with the IncuCyte S3 live cell analysis instrument (Incucyte, Essen BioScience) every 30 min for 4 h. NETosis (excitation at 460 nm and emission at 524 nm) was analyzed by IncuCyte S3 analysis program version 2018B (Essen BioScience).

### Inflammasome Activity Assay

The inflammasome activity was analyzed as described previously [21]. In 500 µL, 0.75 × 10^6^ PBMCs were stimulated with 1 µg/mL LPS for 6 h (Sigma-Aldrich; L3012) and 5 mM ATP for an additional 45 min. The medium was collected after centrifuging the cells 300 g for 5 min at RT and stored at − 80 °C. Enzyme-linked immunosorbent assays (ELISA) were used to measure interleukin (IL)-1β (R&D Systems; DY201) and IL-8 (Fisher Scientific).

### Statistical Analyses

The statistical significance was analyzed with IBM SPSS Statistics 25 software.

## Results

### NFKB1 p.R157X Leads to Reduced Amount of p50 and p105 in PBMCs

**Fig. 2:**
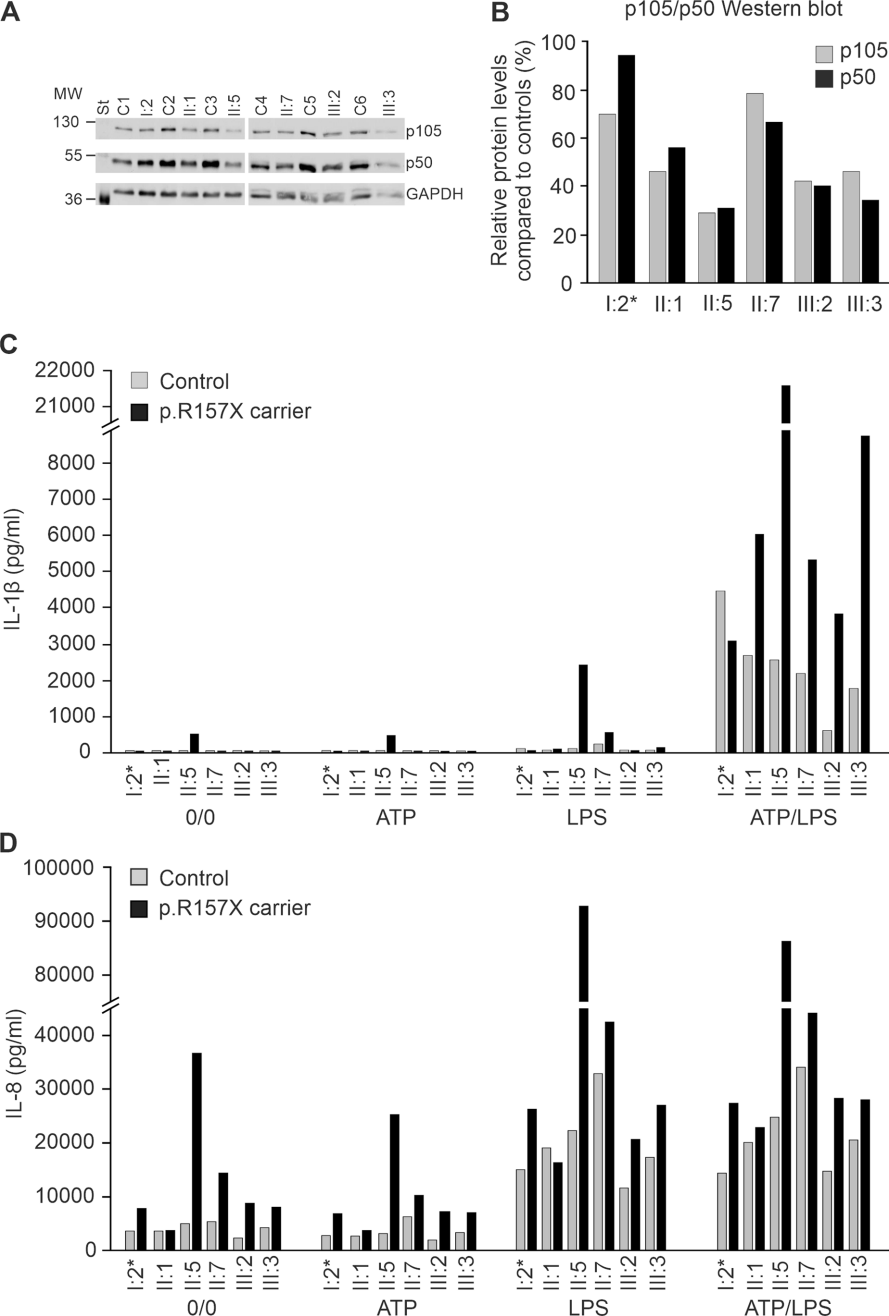
*NFKB1* expression in p.R157X carriers and cytokine analysis. Western blot analysis **A** displays *NFKB1* p50 and p105 subunit levels vs. internal glyceraldehyde 3-phosphate dehydrogenase (GAPDH) control. MW stands for molecular weight standard (St). **B** Quantitation of protein band intensities (patient compared to controls set to 100%) showed that p50 (mean 54%, range 31–94%, *p* < 0.002) and p105 (mean 52%, range 29–79%, *p* < 0.001, Wilcoxon test) were reduced in p.R157X carriers. A single asterisk (*) indicates that age-matched control was not available for the oldest family member (I:2). Interleukin-1β (IL-1β) production is shown in peripheral blood mononuclear cells (PBMC) after stimulation with ATP and/or LPS. **C** Increase in IL-1β level compared to controls (mean 4.4-fold after ATP/LPS stimulation) was observed in *NFKB1* p.R157X carriers (mean 10,706 pg/mL) as compared with healthy controls (mean 2958 pg/mL, *p* = ns, Wilcoxon test). **D** Interleukin-8 (IL-8) production was high in unstimulated *NFKB1* p.R157X positive cases (mean 13,313 pg/mL) compared to controls (mean 4004 pg/mL, *p* < 0.05, Wilcoxon test). IL-8 production in ATP/LPS stimulated *NFKB1* p.R157X PMBCs (mean 39,583 pg/mL, *p* < 0.05) was elevated compared to controls (mean 21,425 pg/mL). A single asterisk (*) indicates that age-matched control was not available for the oldest family member (I:2)

A previous study showed that the p.R157X premature termination of *NFKB1* transcription in the Rel homology domain leads to low amounts of wild-type p50 and p105 in the index case (patient II:5) [13, 22]. In the present study, reduced amounts of p50 (mean 54%, range 31–94%, *p* < 0.002) and p105 (mean 52%, range 29–79%, *p* < 0.001, Wilcoxon test) were found in all tested first-degree *NFKB1* p.R157X mutation carriers, compared to the matching controls (Fig. 2A and B). These results further confirm the LOF effect of the *NFKB1* p.R157X.

### p.R157X has Only Modest Effect on Adaptive Immunity

The tested p.R157X family members had not suffered from infection susceptibility, autoimmunity, or other features of CVID. They have not required IgG substitution. In association with this *NFKB1* LOF variant only modest deviations in lymphocyte maturation or serum immunoglobulin levels were observed (Table 1).

**Table 1:** Blood counts and immunophenotyping of lymphocytes. Absolute counts or percentages of various circulating blood cells and their subsets, serum immunoglobulin concentrations and pneumococcal polysaccharide vaccine (Pneumovax**®**) responses in *NFKB1* p.R157X family members are shown

Patient	I:2	II:1	II:5	II:7	III:2	III:3	Normal range
Age at sampling	86	63	56	54	31	20	
Leukocytes	9.9	11.2	10.4	6.4	9.6	7.7	4.5–13.5 × 10^9^/L
Neutrophils	5.5	5.6	6.1	3.6	5.8	4.4	4.5–13.5 × 10^9^
Lymphocytes	2.8	4.0↑	2.5	1.9	2.7	2.6	1.2–3.5 × 10^9^
Monocytes	1.3↑	1.4↑	1.2↑	0.5	0.9↑	0.5	0.2–0.8 × 10^9^/L
Eosinophils	0.23	0.3	0.49↑	0.3	0.11	0.1	0.1–0.4 × 10^9^/L
Basophils	0.04	0.03	0.04	0.1	0.06	0.0	0–0.1 × 10^9^/L
Thrombocytes	311	286	330	338	334	332	150–360 × 10^9^/L
CD19^+^ B cells	58↓	415	275	237	212	380	80–616/17–26%
Transitional CD38^hi^IgM^hi^	0.6	1.2	3.1	7.9↑	1.8	2.2	0.6–3.5%
Naïve CD27^−^IgD^+^IgM^+^	61.8	90.5↑	85.9↑	93.6↑	73.5	88.2↑	43.2–82.4%
Memory CD27^+^	26.6	8.2	13.2	5.2	24.2	9.7	
Marginal zone CD27^+^IgD^+^IgM^+^	10.8	1.7↓	8.4	2.4↓	19.1	4.4↓	7.2–30.8%
Switched memory CD27^+^IgD^−^IgM^−^	6.4↓	5.4↓	3.8↓	2.4↓	3.9↓	4.7↓	6.5–29.2%
Plasmablasts CD38^++^IgM^−^	< 0.2	< 0.2	< 0.2	< 0.2	< 0.2	< 0.2	0.4–3.6%
Activated CD38^low^CD21^low^	22.7↑	2.3	2.2	2.9	2.8	3.1	0.8–7.7%
CD3^+^ T cells	2641	2901↑	1450	1126	2201	1712	742–2750/56–86%
CD4^+^	1317	971	696	555	1363	977	404–1612/33–58%
CD8^+^	1406↑	1859↑	640	596	773	649	220–1129/13–39%
CD4/CD8	0.9	0.5	1.1	0.9	1.8	1.5	0.6–2.8%
NK	151	415	675	304	371	526	84–724/5–26%
RTE CD45RA^+^CD62L^+^CD31^+^	20.9	17.0	22.3	NA	44.0↑	NA	14.4–38.3%
CD4^+^CD3^+^ T cells							
Naïve CD45RA^+^CCR7^+^	38.9	50.2	60.2↑	NA	82.1↑	NA	20.5–54.8%
TCM CD45RA^−^CCR7^+^	21.3	30.6	29.0	NA	13.3	NA	8.4–32.8%
TEM CD45RA^−^CCR7^−^	38.5	18.7↓	10.5↓	NA	4.4↓	NA	19.9–52.4%
TEMRA CD45RA^+^CCR7^−^	1.3↓	0.5↓	0.4↓	NA	0.2↓	NA	1.4–17.0%
Treg FOXP3^+^CD25^+^	NA	3.8	3.9	NA	4.2	NA	2.0–4.1%
Treg CD127^−^CD25^+^	4.5	6.3	8.5	NA	4.3	NA	3.2–9.3%
Treg in vitro suppression		Normal	Normal				
Th17CD4^+^CD45RA^−^CXCR3^−^CCR6^+^	NA	11.6 ↓	14.6 ↓	10.6 ↓	17.5	14.3 ↓	16–34%
Th17 CD69^+^: IL-17A in CD3/CD28 stimulated cells		0.54 ↓	0.59 ↓				0.76–1.4%
CD8^+^CD3^+^ T cells							
Naïve CD45RA^+^CCR7^+^	4.9↓	19.9	41.6	NA	84.5↑	NA	18.8–71.0%
TCM CD45RA^−^CCR7^+^	7.1	2.7	6.2	NA	4.2	NA	1.2–7.3%
TEM CD45RA^−^CCR7^−^	36.4	7.7↓	11.0↓	NA	7.5↓	NA	14.6–63.0%
TEMRA CD45RA^+^CCR7^−^	51.6↑	69.8↑	41.3 ↑	NA	3.9	NA	4.5–33.7%
Immunoglobulins							
S-IgG	4.6↓	6.4↓	8.7	7.5	5.2↓	7.3	6.77–15 g/L
S-IgA	0.94	2.17	1.24	1.05	0.79↓	1.05	0.88–4.84 g/L
S-IgM	0.36	0.22↓	0.76	0.19↓	0.42	0.28↓	0.36–2.59 g/L
S-IgE	5	24	23	16	9	8	0–130 IU/L
S-IgG1	NA	5.6	NA	4.67	NA	5.23	4.05–10.11 g/L
S-IgG2	NA	2.7	NA	2.82	NA	1.68↓	1.69–7.86 g/L
S-IgG3	NA	0.13	NA	0.22	NA	0.17	0.11.–0.85 g/L
S-IgG4	NA	0.13	NA	0.86	NA	1.3	0.03–2.01 g/L
Vaccine response							
Pneumovax	NA	NA	NA	6/10	4/10	9/10	

### IL-1β Production is High in p.R157X PBMCs

NF-κB signaling primes the NLRP3 inflammasome for activation by inducing the expression of NLRP3 as well as pre-IL-1β. Previously, we have shown that macrophages of II:1 and II:5 produce high amounts of IL-1β in vitro [13]. As expected, the PBMCs of the index brothers, and the first-degree p.157X mutation carriers, showed increased IL-1β production after ATP/LPS stimulation (Fig. 2C) compared to controls (mean 4.4-fold increase,* p* < 0.05, Wilcoxon test). The most pronounced IL-1β levels were observed in the index patient (II:5) (Fig. 2C).

### Elevated IL-8 Production in p.R157X PBMCs

Very high acute phase blood neutrophil counts (68.0 × 10^9^/L) during necrotizing fasciitis prompted us to measure the production of IL-8, an important cytokine for neutrophil survival and chemoattraction, in stimulated PBMCs. IL-8 is also known to stimulate neutrophil mobilization and viability, potentially additionally driven by IL-1β [23, 24]. We found that IL-8 production in p.R157X mutation carriers was increased in vitro (Fig. 2D), not only after ATP/LPS stimulation (39 583 pg/mL vs. 21 425 pg/mL, *p* < 0.05, Wilcoxon test) but also under resting conditions (13,313 pg/mL vs. 4004 pg/mL, *p* < 0.05, Wilcoxon test). The most pronounced IL-8 levels were observed in the index patient (II:5) (Fig. 2D).

### Defective NF-κΒ Signaling in p.R157X Neutrophils

**Fig. 3:**
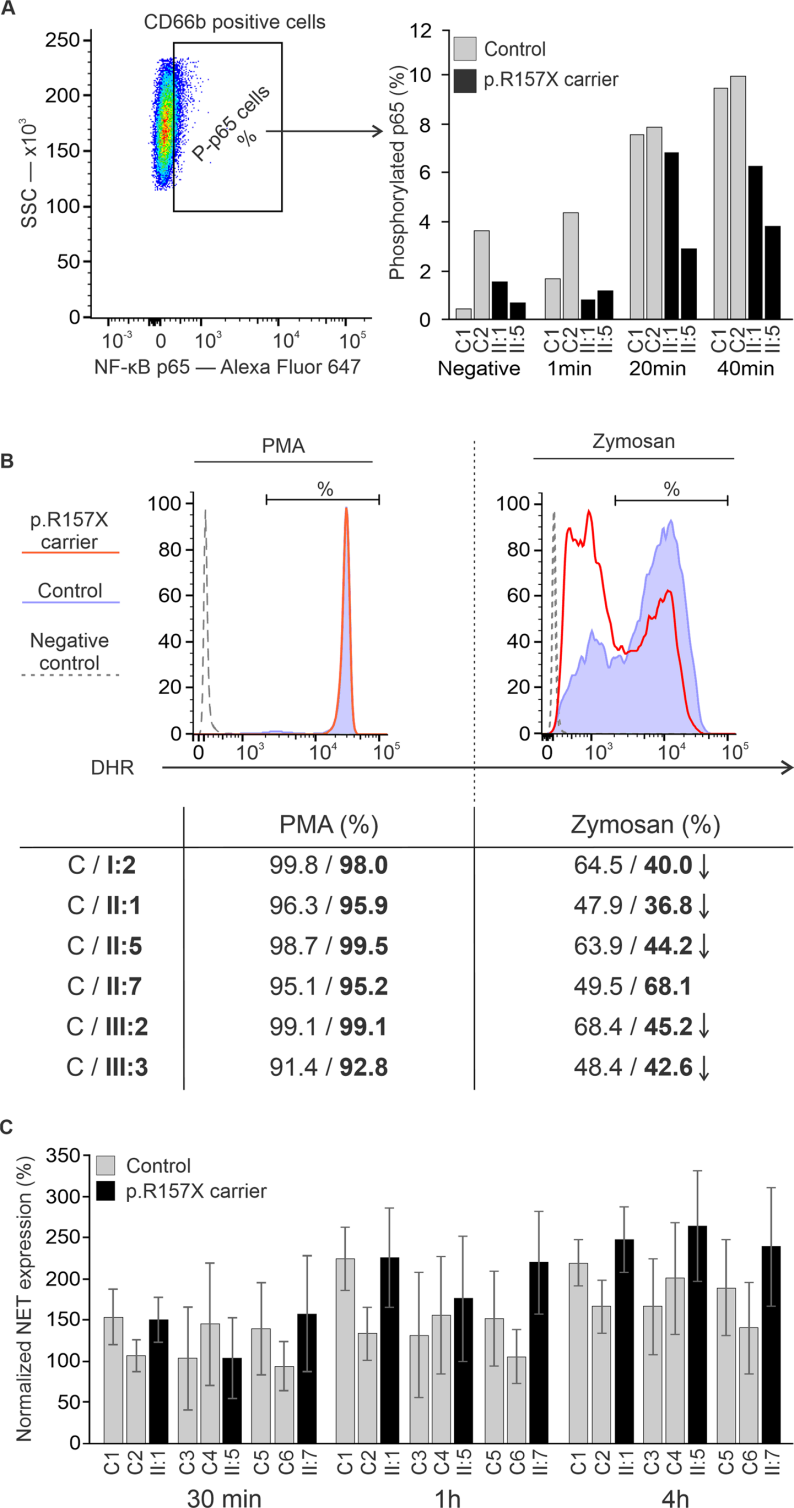
Neutrophil analysis. **A** NF-κΒ pathway was stimulated with zymosan (1 min, 20 min and 40 min, in duplicates) in *NFKB1* p.R157X (patients II:1 and II:5) and healthy control neutrophils and measured for phosphorylated p65/RelA. The average percentage of activated neutrophils positive for RelA/p65 phosphorylation was low in patients’ (II:1 and II:5) (5.0%) neutrophils compared to controls (9.7%) after 40-min stimulation. **B** Dihydrorhodamine (DHR) test showed robust activation in *NFKB1* p.R157X (96.8%) and control neutrophils (97.0%) after PMA stimulation. Representative results of DHR assay (patient II:5) for highly stimulated *NFKB1* p.R157X neutrophils compared to control neutrophils after zymosan stimulation. Mean percentage in the *NFKB1* p.R157X neutrophils was (46.7%) compared to controls (56.3%, *p* < 0.05). **C** Neutrophil extracellular trap formation was similar in *NFKB1* p.R157X neutrophils compared to controls. Values are averages of eight replicates normalized to corresponding samples without stimulation at starting points. Error bars present standard deviation

The brothers (II:1, II:5) with necrotizing fasciitis presented with very high neutrophil counts. Neutrophils play a significant role in the pathogenesis and tissue destruction in infectious fasciitis [25]. Therefore, we explored further the impact of *NFKB1* p.R157X mutation on the function of neutrophils. Dectin-1 and TLR2 stimulation activates spleen tyrosine kinase (Syk) and caspase recruitment domain family member 9 (CARD9). These events are followed by NF-κΒ activation and generation of ROS [17, 26–29]. Neutrophils were stimulated with zymosan, a fungal component, which triggers the ROS production, and has been used in diagnostic procedures for chronic granulomatous disease (CGD) [30]. A significant increase in RelA/p65 phosphorylation was observed after zymosan stimulation in control neutrophils, whereas in p.R157X neutrophils phosphorylation of RelA/p65 was compromised (Fig. 3A, Supplemental Fig. 1).

### Oxidative Burst and NETosis in NFKB1 p.R157X

In the NBT test, chemiluminescence after zymosan stimulation was consistent with defective oxidative burst in the neutrophils of p.R157X fasciitis brothers II:1 (289 mV) and II:5 (237 mV) when compared with a variant negative sibling II:2 (1548 mV) [18, 19]. To further assess the impact of *NFKB1* p.R157X on the neutrophil function, we stimulated the neutrophils with PMA, an NF-κΒ-independent activator, and studied neutrophil responses using the DHR test. Robust oxidative burst response was observed both in controls (96.8%) and in p.R157X variants (97.0%) (Fig. 3B). The results confirmed the NADPH complex to be intact in the p.R157X neutrophils. However, the percentage of neutrophils with highly activated oxidative bursts was lower in *NFKB1* p.R157X neutrophils compared to controls after stimulation of NF-κB-dependent TLR2 and Dectin-1 receptors with zymosan (Fig. 3B). This was especially noticeable among male patients (*p* < 0.05, paired samples *t*-test). Oxidative burst in monocytes could not be evaluated because of their similar positioning with zymosan particles in flow cytometric 2-D scatter plot. iNETosis in the p.R157X neutrophils was comparable to healthy controls after both zymosan (Fig. 3C) and PMA stimulation (Supplemental Fig. 4 and 5).

### NADPH Oxidase Subunit Quantitation

We considered the possibility that the defective oxidative burst caused by *NFKB1* p.R157X variant can be explained by the expression of NADPH oxidase subunits or quantity of their gene products in neutrophils. The Western blots of NADPH complex subunits (gp91^phox^, p22^phox^, p40^phox^, p47^phox^, p67^phox^, RAC2) were comparable in p.R157X and control neutrophils (Supplemental Fig. 2). The NADPH oxidase complex subunit mRNA expression levels were equal both in p.R157X and control neutrophils in unstimulated conditions (Supplemental Fig. 3). Under zymosan stimulation, they were below the accurate quantitation limit in control and p.R157X neutrophils (Supplemental Fig. 3).

## Discussion

Family members of this report suffered from recurrent necrotizing fasciitis or intense wound inflammation in association with heterozygous *NFKB1* p.R157X variant. Consistent with our original observation, similar clinical presentations in patients with *NFKB1* LOF variants have been reported [14–16]. In conclusion, sterile necrotizing fasciitis or neutrophilic dermatoses are associated with inborn errors of immunity [31, 32, [33]. In the present study, microbiological cultures collected during the fasciitis episodes remained negative; CRP levels were high and neutrophil counts exceptionally so, without clinically evident responses to antibiotics. However, our patients seemed to benefit from systemic corticosteroids and the IL-1 receptor antagonist anakinra. Overall, the clinical presentations were consistent with exaggerated immune reaction after perioperative tissue trauma rather than aggressive infection. However, with thus far used methods here and in literature, aberrant adaptive immunity in *NFKB1* pathogenic mutation carriers with fasciitis has not appeared to contribute to the development of necrotizing fasciitis.

Elevated inflammatory cytokine levels were found not only in the index brothers but also in asymptomatic *NFKB1* p.R157X mutation carriers in the family. Blood neutrophil counts during fasciitis episodes were exceptionally high, up to 68 × 10^9^/L (normal range 4.5–13.5 × 10^9^/L). We hypothesize that the high IL-1β and IL-8 levels during immune activation may contribute to such blood neutrophil levels. Carrier neutrophils were also affected by the *NFKB1* p.R157X LOF, since NF-κB-dependent pathways failed to activate RelA/p65 phosphorylation and proper oxidative burst. However, oxidative burst appeared normal in response to NF-κB-independent PMA stimulus. The results of our NADPH oxidase complex subunit quantitation in p.R157X neutrophils were comparable to controls, although minor changes in the complex subunits cannot be excluded by Western blotting [5, 34]. We can only speculate that the *NFKB1* p.R157X LOF variant may selectively disturb the instant events such as phosphorylation of subunits, protein–protein interactions, protein-lipid interactions, or properties of energy metabolism involved in the carefully regulated oxidative burst [35].

We believe that defects in neutrophils may contribute to the observed clinical consequences due to their increased numbers in blood and potentially in affected tissues. Despite the low p50 and p105 levels, significantly increased activity of NLRP3 inflammasome and defective oxidative burst, the mutation carriers’ clinical phenotype is characterized by long quiescent asymptomatic periods and absence of inflammatory periods. Interestingly, patients with X-linked chronic granulomatous disease (CGD) (gp91^phox^ deficiency) share biological properties such as low p65 phosphorylation, defective ROS production, and high inflammasome activity with our p.R157X cases [36]. However, p.R157X carriers have not suffered from fungal infections, which are common in X-linked CGD. Although the p.R157X patients experienced periods of severe skin and soft tissue inflammation, no systemic autoinflammatory symptoms commonly seen in other conditions causing activation of canonical inflammasome could be observed, suggesting that additional concurrent stimuli are needed [13, 37].

In conclusion, *NFKB1* LOF variants can be associated with diverse clinical and immunological presentations [12, 13]. In the family described here, the carriers of the *NFKB1* p.R157X LOF demonstrated significantly increased responsiveness to immune activation. Despite this, some carriers have remained asymptomatic, whereas others experienced episodes of necrotizing fasciitis. This suggests that additional immunological triggers such as trauma or operation are needed for disease onset. Our results also demonstrate that the variant affects the NF-κΒ-dependent activation pathways in neutrophils, and thus implicate that the neutrophils may have a significant role in the disease manifestations in sterile necrotizing fasciitis.

## Supplementary Information

Below is the link to the electronic supplementary material.

Supplementary file1 (DOCX 1396 KB)

Supplementary file2 (DOCX 17 KB)

## Data Availability

Available upon reasonable request to the corresponding authors.
