# Supplementary material for: Inflammation and Neutrophil Oxidative Burst in a Family with NFKB1 p.R157X LOF and Sterile Necrotizing Fasciitis
Source: J Clin Immunol. 2023 Mar 9;43(5):1007–18. doi: 10.1007/s10875-023-01461-3 (PMC10276129; doi:10.1007/s10875-023-01461-3)
Supplement: Supplementary file 1 — Supplementary file1 (DOCX 1396 KB) [file 10875_2023_1461_MOESM1_ESM.docx]

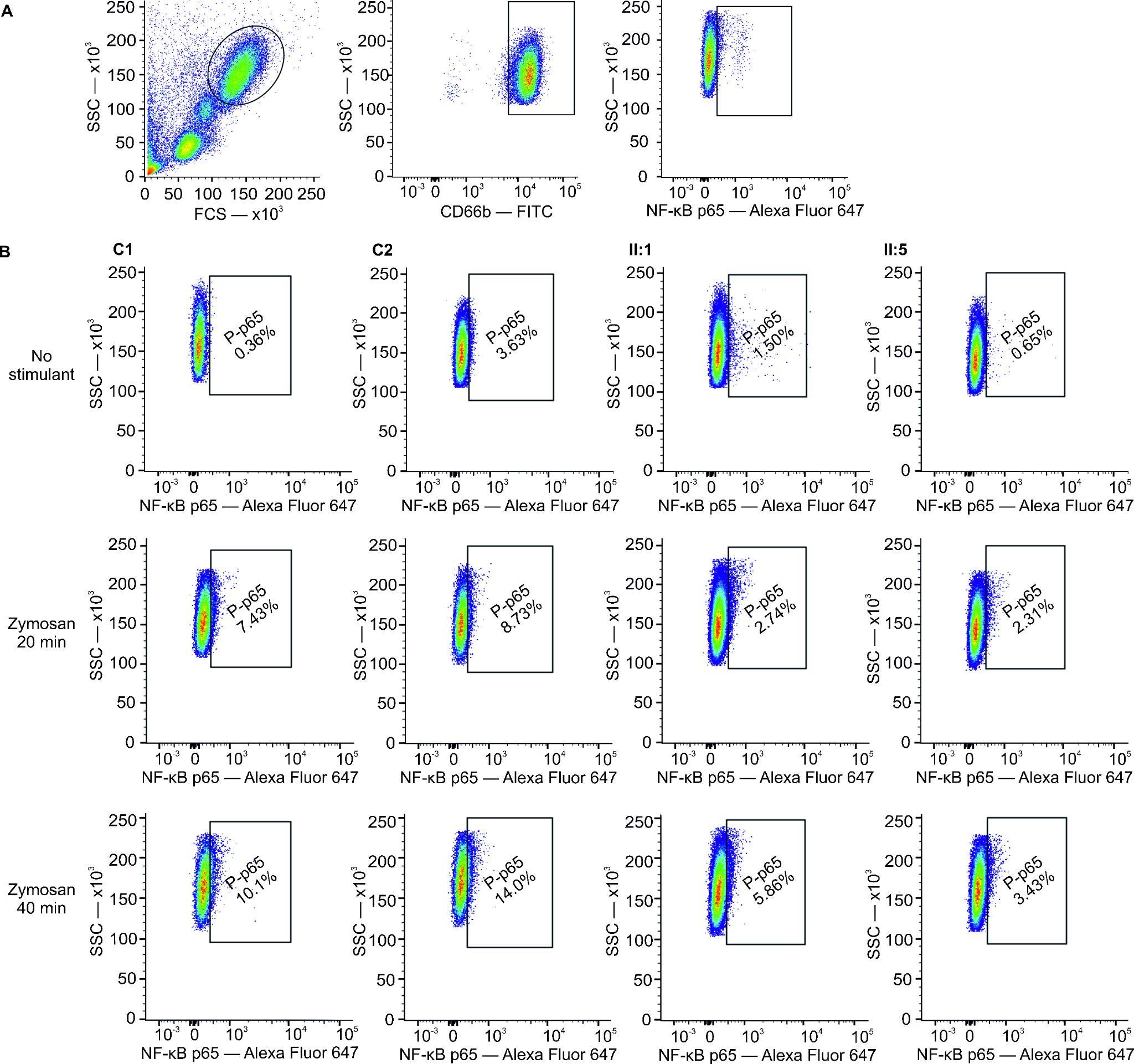


**Supplemental Figure 1.** Gating strategy (A) and percentages of phosphorylated p65 in CD66b positive neutrophils after whole blood zymosan stimulation (B). The results are representatives from controls (C1 and C2) and *NFKB1* p.R157X variant positive patients with necrotizing fasciitis (II:1 and II:5) in three timepoints (non-stimulated, 20 minutes and 40 minutes).


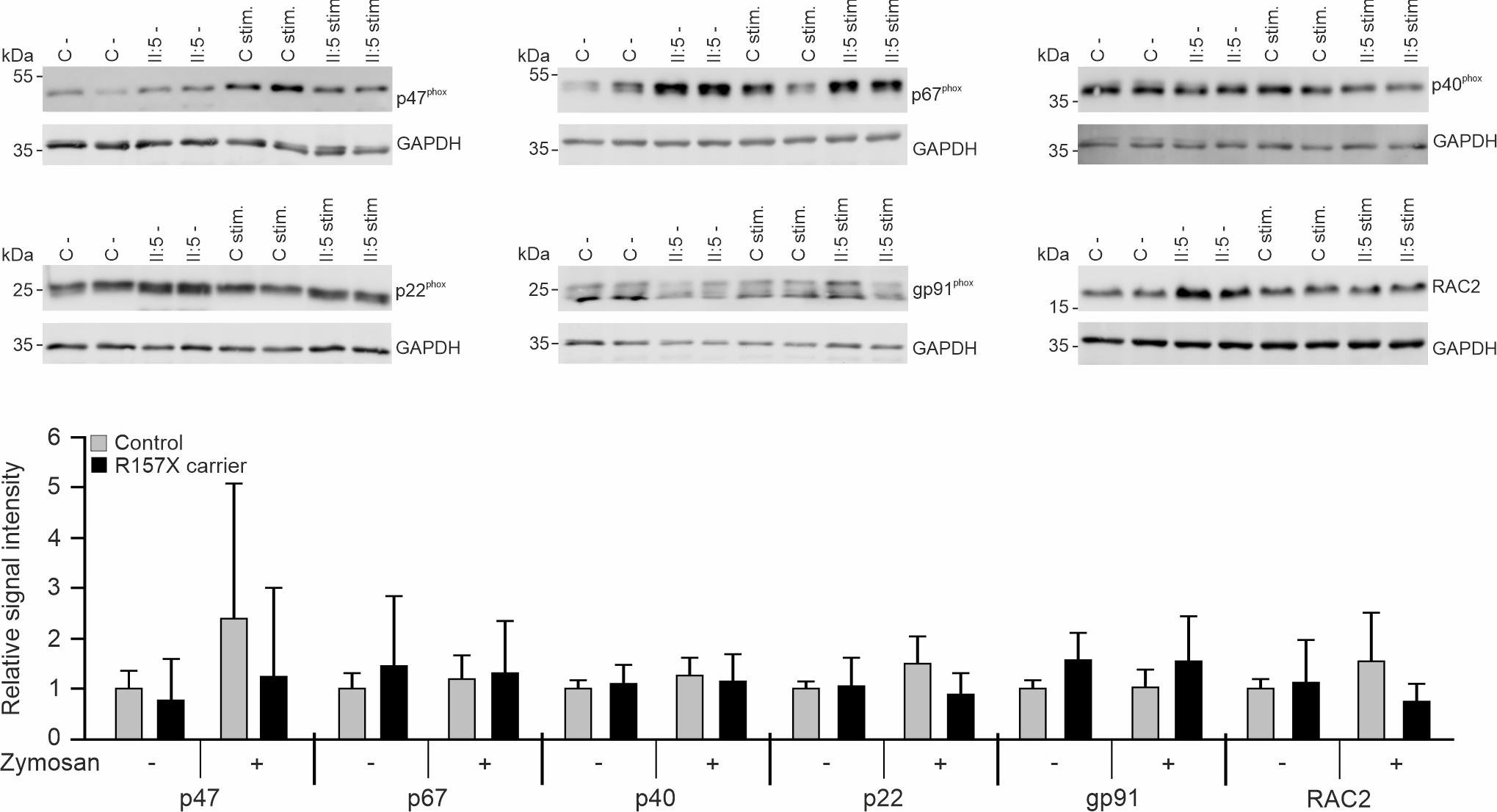


**Supplemental Figure 2.** Presentative western blot analysis of NADPH complex subunits (p47^phox^, p67^phox^, p40^phox^, p22^phox^, gp91^phox^, RAC2) in the patient and control neutrophils before and after zymosan stimulation. Bar chart is presentative of protein levels relative to the unstimulated control samples. Values are averages from three separated Western blots from two mutation carriers.


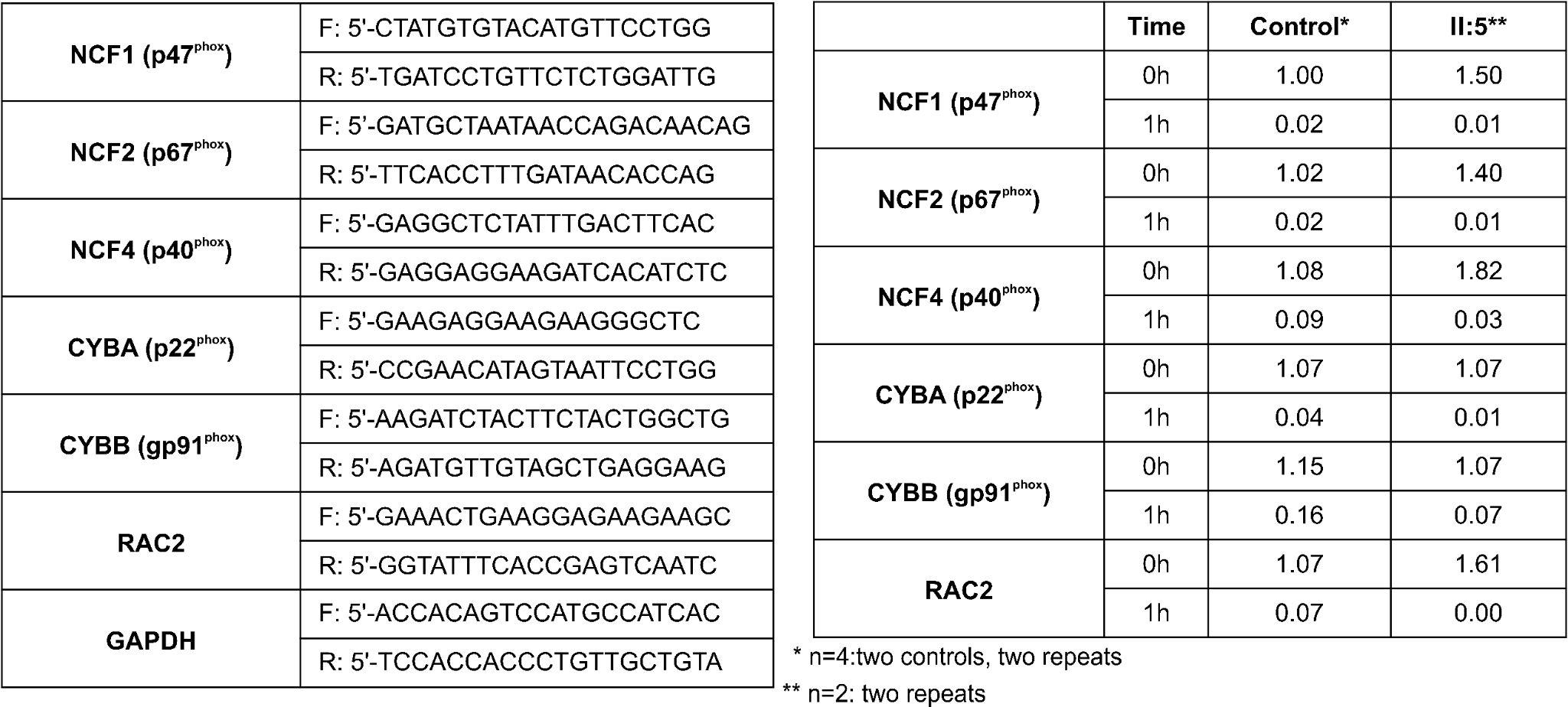


**Supplemental Figure 3.** NADPH complex subunit (p47^phox^, p67^phox^, p40^phox^, p22^phox^, gp91^phox^, RAC2) primers (left) and the qPCR analysis of the subunits’ expression in the *NFKB1* p.R157X patient II:5 and control neutrophils before and after 1 hour after zymosan stimulation (right). Values are averages from duplicates on two separated runs and normalized to housekeeping gene (GAPDH) with ΔΔCt method. N for controls was 4 (2 volunteers, 2 runs) while patient samples were from II:5 (n:2).


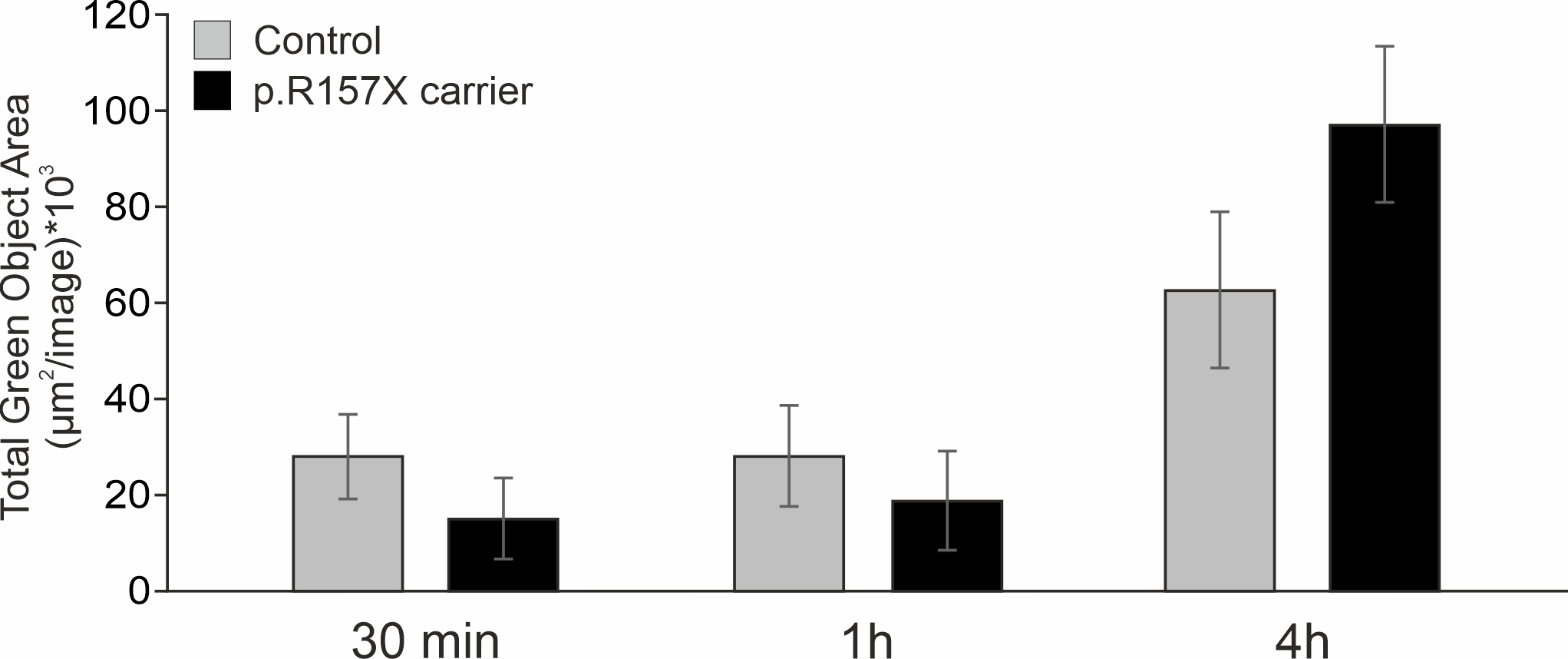


**Supplemental Figure 4.** A presentative figure of PMA stimulated NETosis in control and p.R157X carrier neutrophils.

**
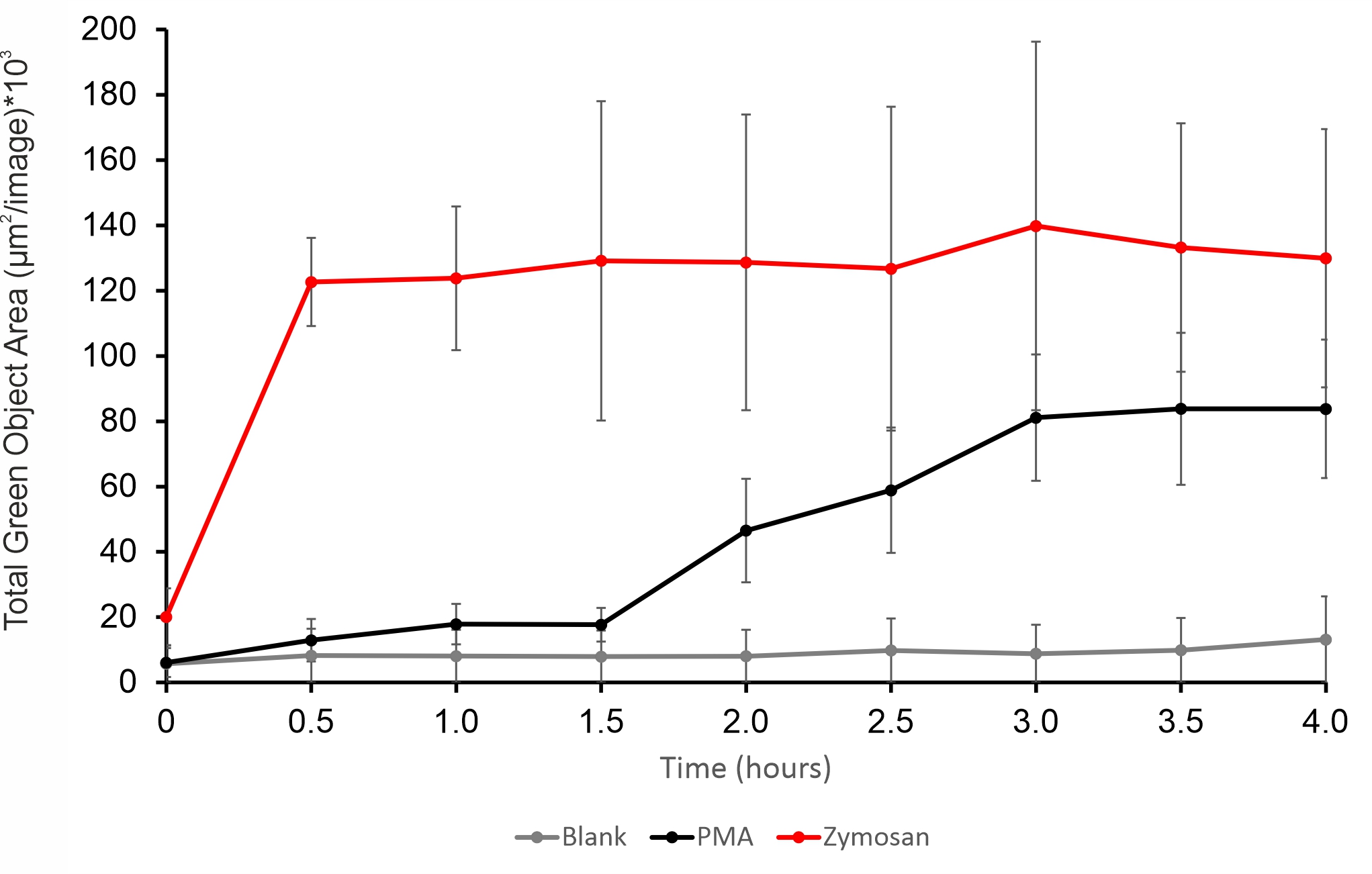
**

**Supplemental Figure 5.** Comparison of PMA (100nM) and zymosan (50 µg/mL) as NETosis stimulants. The experiment was performed with control neutrophils to confirm stimulants’ function before collection of the patient samples.
