# Supplementary material for: Inflammation and Neutrophil Oxidative Burst in a Family with NFKB1 p.R157X LOF and Sterile Necrotizing Fasciitis
Source: J Clin Immunol. 2023 Mar 9;43(5):1007–18. doi: 10.1007/s10875-023-01461-3 (PMC10276129; doi:10.1007/s10875-023-01461-3)
Supplement: Supplementary file 2 — Supplementary file2 (DOCX 17 KB) [file 10875_2023_1461_MOESM2_ESM.docx]

**Santaniemi et al, Supplement methods**

**Protein extraction from neutrophils**

Neutrophils were isolated as described from patient II:5 and matching control. The cells were counted, suspended to final concentration of 6 x 10^6^ cells/mL in medium (RPMI medium containing 2 mM L-Glutamine) and split into four tubes each containing 2 mL of cell suspension. Zymosan was added in two tubes for final concentration of 50 x 10^3^ particles/µL (138 µg/mL) for 1 hour at RT. The cells were washed twice with PBS before adding 400 µL of lysis buffer (2x inhibitors). The protein concentration was determined with DC protein assay (BioRad).

**Western blot analysis of NADPH complex subunits**

20µg of protein was mixed in Laemmli sample buffer (BioRad, #1610747) with β-mercaptoethanol (5%). Western blot was performed as described after separating the samples on 10% (gp91phox, p67phox, p47phox and p40phox) or 15% (p22phox, RAC2) SDS-PAGE gels. The membranes were blocked with 5% milk in TBS-T or blocking buffer (LICOR) and the antibodies bound on membranes overnight at 4 °C [2 µg/mL rabbit-anti p67phox (ab109523), rabbit-anti p47phox (ab137950), RAC2 (ab130415) 0.1 µg/mL mouse-anti p40phox (ab76158), Abcam. 2 µg/mL mouse-anti p22phox (MCA4686), mouse-anti gp91phox (MCA4685), BioRad AbD Serotec. 2 µg/mL rabbit phospho-p47phox (Ser345) (PA5-36863) and 1 µg/mL mouse anti-GAPDH (Clone 258), Thermo Fisher Scientific]. After washing thrice for 15 minutes with TBS-T, 1:10 000 of Goat Anti-Mouse IgG(H+L) Human ads-HRP (1031-05 AH Diagnostics), Goat Anti-Rabbit IgG H&L (HRP) preadsorbed (Abcam, ab97080), mouse IRDye 680RD or 800CW (LI-COR) secondary antibodies were added for 45 minutes at RT. After washing as above, the membranes were imaged with Odyssey infrared scanner (LI-COR) or after adding WesternBright ECL-spray (Advansta, #K-12049-D50) for 2 minutes on both sides, with Azure 600 imager (Azure Biosystems). The intensities were analyzed by Image Studio Lite Ver 5.2 (LI-COR).

**RNA extraction from neutrophils and cDNA synthesis**

Neutrophils were isolated as before from patient II:5 and two control subjects. The cells were plated on a 6-well plate, 20 x 10^6^ cells / 1.6mL (RPMI medium containing 2 mM L-Glutamine). Zymosan was added to a final concentration of 50 x 10^3^ particles/µL for 1h at RT. The cells with and without zymosan were pelleted (500g, 5min), resuspended in 300 µL of RNAProtect Cell reagent (Qiagen, REF: 76526) and stored at -80°C.

The RNA was isolated by using the RNeasy Mini Kit (Qiagen, REF: 74106). cDNA was created by using RevertAid First Strand cDNA Synthesis Kit with DNase I (EN0521 and K1622; Thermofisher).

**qPCR analysis of NADPH complex subunits**

qPCR was performed with FastStart Universal SYBR Green Master (Rox) (Merck, REF: 4913850001). The primers (Sigma-Aldrich) are listed in Supplement Figure 3. GAPDH (Eurogentec) was used as a housekeeping gene. The samples were subjected to QuantStudio® 5 System and Comparative-Ct-SYBR method and the data analyzed with DataConnect (Thermofisher) and Microsoft Excel by using the 2^-ΔΔCt^ method.
